# LncLocFormer: a Transformer-based deep learning model for multi-label lncRNA subcellular localization prediction by using localization-specific attention mechanism

**DOI:** 10.1093/bioinformatics/btad752

**Published:** 2023-12-18

**Authors:** Min Zeng, Yifan Wu, Yiming Li, Rui Yin, Chengqian Lu, Junwen Duan, Min Li

**Affiliations:** School of Computer Science and Engineering, Central South University, Changsha, Hunan 410083, China; School of Computer Science and Engineering, Central South University, Changsha, Hunan 410083, China; School of Computer Science and Engineering, Central South University, Changsha, Hunan 410083, China; Department of Health Outcomes and Biomedical Informatics, University of Florida, Gainesville, FL 32603, United States; School of Computer Science, Key Laboratory of Intelligent Computing and Information Processing, Xiangtan University, Xiangtan, Hunan 411105, China; School of Computer Science and Engineering, Central South University, Changsha, Hunan 410083, China; School of Computer Science and Engineering, Central South University, Changsha, Hunan 410083, China

## Abstract

**Motivation:**

There is mounting evidence that the subcellular localization of lncRNAs can provide valuable insights into their biological functions. In the real world of transcriptomes, lncRNAs are usually localized in multiple subcellular localizations. Furthermore, lncRNAs have specific localization patterns for different subcellular localizations. Although several computational methods have been developed to predict the subcellular localization of lncRNAs, few of them are designed for lncRNAs that have multiple subcellular localizations, and none of them take motif specificity into consideration.

**Results:**

In this study, we proposed a novel deep learning model, called LncLocFormer, which uses only lncRNA sequences to predict multi-label lncRNA subcellular localization. LncLocFormer utilizes eight Transformer blocks to model long-range dependencies within the lncRNA sequence and shares information across the lncRNA sequence. To exploit the relationship between different subcellular localizations and find distinct localization patterns for different subcellular localizations, LncLocFormer employs a localization-specific attention mechanism. The results demonstrate that LncLocFormer outperforms existing state-of-the-art predictors on the hold-out test set. Furthermore, we conducted a motif analysis and found LncLocFormer can capture known motifs. Ablation studies confirmed the contribution of the localization-specific attention mechanism in improving the prediction performance.

**Availability and implementation:**

The LncLocFormer web server is available at http://csuligroup.com:9000/LncLocFormer. The source code can be obtained from https://github.com/CSUBioGroup/LncLocFormer.

## 1 Introduction

Long non-coding RNAs (lncRNAs) are a class of non-coding RNA molecules that comprised more than 200 nucleotides (Birney *et al.* 2007, Lu *et al.* 2018). They are involved in various important biological processes, including the regulation of gene expression, alternative splicing, nuclear organization, and genomic imprinting (Zeng *et al.* 2020). LncRNAs have the ability to interact with proteins, DNAs, and RNAs, and perform specific functions as a result of these interactions (Esteller 2011). For example, they can act as “miRNA sponge” to regulate miRNA levels and thereby influence the expression of miRNA targets (DiStefano 2018). Additionally, under particular stimulation, lncRNAs can influence transcriptional activity or pathways (Wang and Chang 2011). Because of the complexity of molecular functions and biological processes, lncRNA-related research has gained significant attention (Lu *et al.* 2020, Zeng *et al.* 2021).

A growing amount of evidence reveals that lncRNA subcellular localizations can provide valuable insights into their biological functions (Savulescu *et al.* 2021). One wet-lab technique commonly used to study RNA subcellular localization is single-molecule fluorescent *in situ* hybridization (smFISH) technique (Moffitt and Zhuang 2016). Although the image data provided by the smFISH technique can accurately determine the subcellular localization of RNAs, the smFISH technique is expensive and time-consuming. Considering its limitations, it would be extremely beneficial for biologists to develop accurate computational methods to predict lncRNA subcellular localizations.

Some computational methods have been proposed to predict lncRNA subcellular localization. To the best of our knowledge, LncLocator is the first predictor for lncRNA subcellular localization (Cao *et al.* 2018). It extracts 4-mer features and high-level features, and uses support vector machine (SVM) and random forest to make predictions. iLoc-lncRNA utilizes 8-mer features to encode lncRNA sequences, and applies SVM to perform the prediction task (Su *et al.* 2018). DeepLncRNA incorporates 2, 3, 4, and 5-mer features and uses a deep learning network to predict lncRNA subcellular localizations (Gudenas and Wang 2018). Locate-R incorporates the preselected *k*-mer features and applies SVM to construct a classifier (Ahmad *et al.* 2020). lncLocPred integrates multiple feature selection techniques to select optimal features, and adopts a logistic regression model to make predictions (Fan *et al.* 2020). LncLocation integrates the multi-source heterogeneous features, and uses SVM to construct a classifier (Feng *et al.* 2020). DeepLncLoc is a novel deep learning model, which uses subsequence embedding technique to encode lncRNA sequences, and uses a deep neural network to classify five localizations (Zeng *et al.* 2022). TACOS applies a tree-based stacking classifier to predict the subcellular localization of human lncRNA in 10 different cell types (Jeon *et al.* 2022). RNALight extracts *k*-mer features and uses LightGBM to predict the subcellular localizations of mRNAs and lncRNAs (Yuan *et al.* 2023). GraphLncLoc transforms lncRNA sequences into graphs, and utilizes graph convolutional networks to capture high-level features and make predictions (Li *et al.* 2023). Recently, LncLocator 2.0 (Lin *et al.* 2021) and iLoc-LncRNA(2.0) (Zhang *et al.* 2022) have been released, which provide more accurate prediction results than their previous versions.

Although several computational models have been developed, few of these models are designed for lncRNAs that have multiple subcellular localizations. In reality, lncRNA subcellular localization is a dynamic process (Bridges *et al.* 2021). For example, lncRNA SNHG1 displays cytoplasmic distribution in human HCT116 colon cancer cells. However, upon DNA damage stress, it is retained in the nucleus compartment (Carlevaro-Fita and Johnson 2019). Another example is lncRNA Uchl1-AS1, which translocates from the nucleus to the cytoplasm under rapamycin treatment (Riva *et al.* 2016). However, the existing computational models usually only consider a single subcellular localization for each lncRNA.

In addition, increasing evidence suggests that lncRNAs exhibit distinct localization patterns in different subcellular localizations. For example, Shukla *et al.* found that conserved long sequences (>300 nt) with a common 15-nt C-rich pattern are responsible for nuclear localization (Shukla *et al.* 2018). Lubelsky *et al.* found a core 42-nt motif that drives nuclear RNA localization (Lubelsky and Ulitsky 2018). Despite these findings, existing computational methods do not take motif specificity in different subcellular localizations into account.

To meet the need for lncRNA multiple subcellular localization predictions and to consider motif specificity for different subcellular localizations, we proposed LncLocFormer, which is a Transformer-based deep learning model using a localization-specific attention mechanism. Transformer is a class of powerful deep learning architecture that has achieved substantial breakthroughs in natural language processing (NLP), as it can capture both local and global features of sequences. Inspired by its success in NLP, we applied it to the prediction of lncRNA subcellular localization. By using the positional coding and multi-head attention mechanism in Transformer blocks, LncLocFormer can model long-range dependencies within the lncRNA sequence and share information across the lncRNA sequence. Different from previous computational methods, LncLocFormer can predict multiple subcellular localizations simultaneously for each lncRNA sequence. Furthermore, using the localization-specific attention mechanism, LncLocFormer learns different attention weights for different subcellular localizations, which can provide valuable information about the relationship between different labels.

To evaluate the performance of LncLocFormer, we compared it with some deep learning baseline models and existing state-of-the-art predictors. The results of cross-validation (CV) and the hold-out test set demonstrate that LncLocFormer performs significantly better than other computational models. In addition, the results show that LncLocFormer is capable of capturing sequence motifs. To investigate which part of LncLocFormer is helpful in predicting lncRNA subcellular localizations, we conducted an ablation study by removing or replacing some components of LncLocFormer. The ablation study shows that the localization-specific attention mechanism is a crucial component in LncLocFormer. To facilitate the use of LncLocFormer, we developed a user-friendly web server.

## 2 Materials and methods

### 2.1 Benchmark dataset

The first important step in constructing a reliable predictor is to establish a reliable benchmark dataset. To achieve this, we retrieved known lncRNA subcellular localization information from the RNALocate v2.0 (Cui *et al.* 2022) database (https://www.rna-society.org/rnalocate/), which collects more than 210 000 RNA-associated subcellular localization entries with experimental evidence, encompassing more than 110 000 RNAs with 171 subcellular localizations in 104 species. We generated a benchmark dataset to train and test our model by the following procedure:

1. We retrieved a total of 9128 Homo sapiens lncRNA-associated subcellular localization entries from the RNALocate v2.0 database. Since many lncRNAs have multiple entries, we merged the entries with the same gene symbol;
2. We removed the lncRNAs that do not have sequence information in NCBI (Pruitt *et al.* 2007);
3. To reduce data redundancy, we used the cd-hit-est tool (Huang *et al.* 2010) with a cutoff of 80%;
4. Consider that some subcellular localizations have a small number lncRNA entries, we only selected the subcellular localizations with more than 40 lncRNA entries;
5. In the RNALocate v2.0 database, a significant number of entries are localized in exosome. However, accumulating evidence suggests that lncRNAs are expressed in a cell-specific and/or tissue-specific manner, and most of them are located in the nucleus. Moreover, a lot of samples which belong to the exosome localization could hinder the prediction of other subcellular locations. Thus, we removed exosome-localized entries in our study.

**Figure 1.**
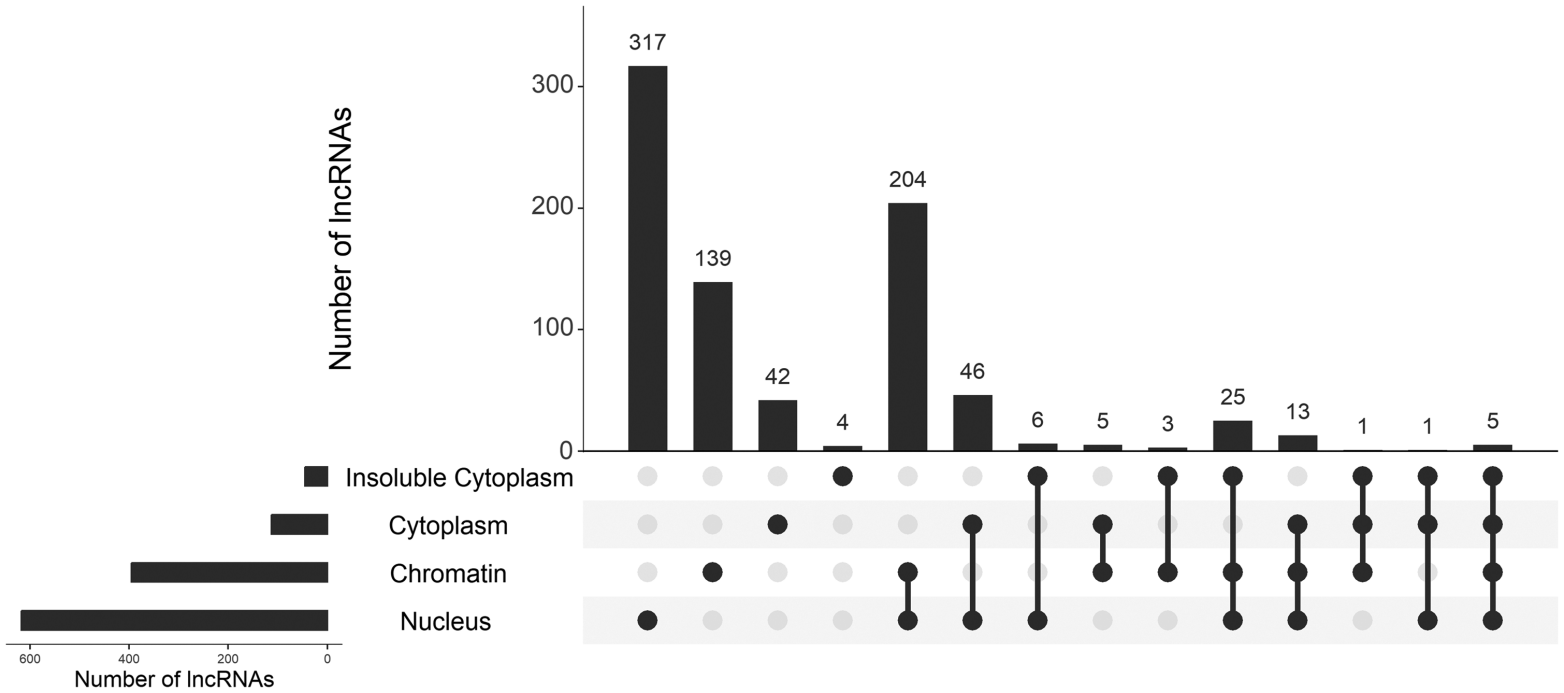
The distribution of subcellular localizations in the constructed benchmark dataset.

Finally, our benchmark dataset comprises 811 lncRNAs, covering four types of subcellular localizations: nucleus, cytoplasm, chromatin, and insoluble cytoplasm. Figure 1 shows the distribution of subcellular localizations in the constructed benchmark dataset.

### 2.2 LncLocFormer architecture

**Figure 2.**
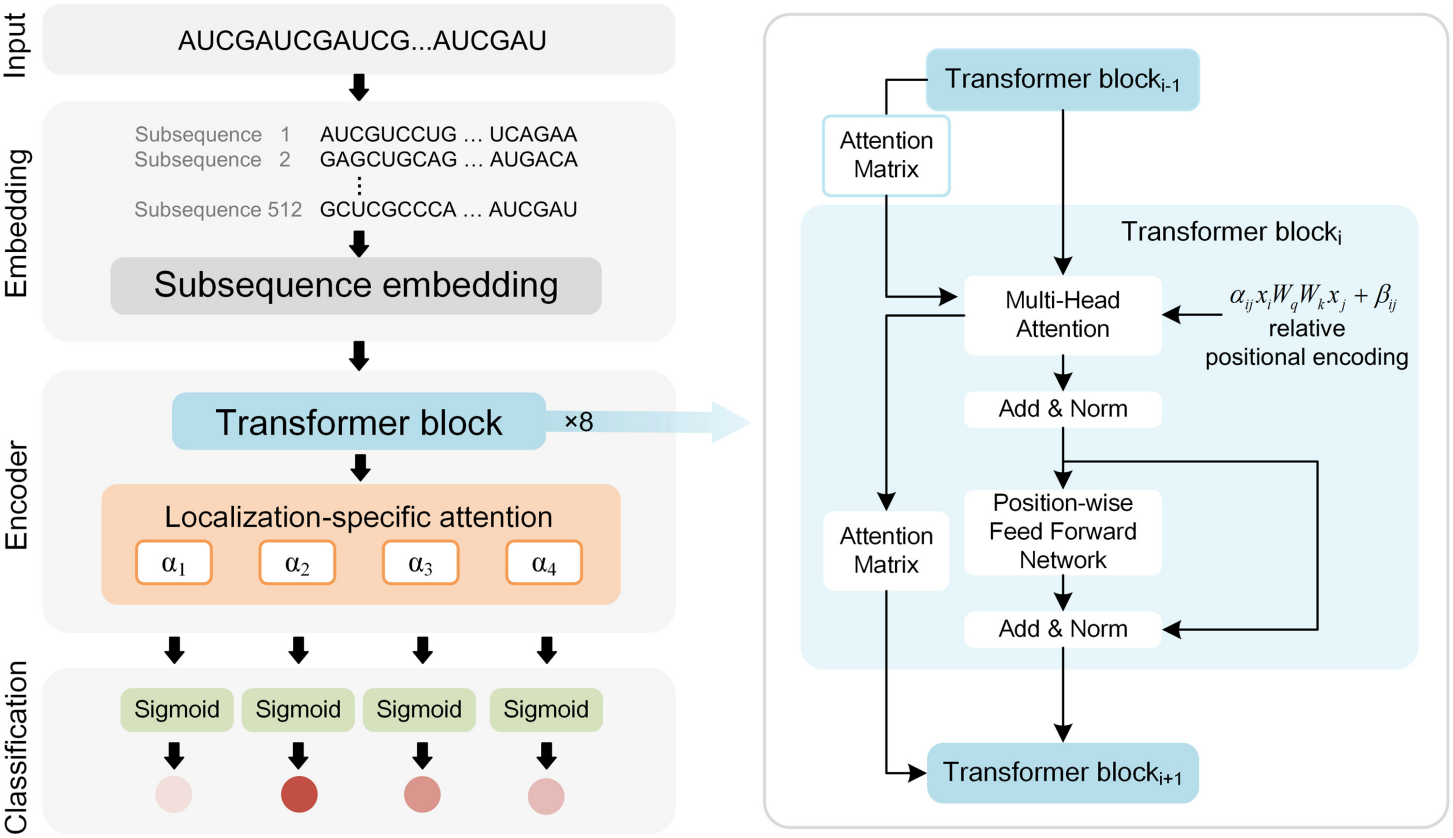
The architecture of LncLocFormer. LncLocFormer takes a lncRNA sequence as input, which is encoded using a subsequence embedding method. The embedding layer is immediately followed by eight Transformer blocks, which are used to model long-range dependencies within the lncRNA sequence and share information across the lncRNA sequence. These Transformer blocks have the same architecture: relative position encoding, multi-head attention mechanism, residual connection, position-wise feed-forward network, and “Add & Norm” component. Following the Transformer blocks, the designed localization-specific attention layer is employed to learn distinct weights of nucleotide for each subcellular localization. Finally, a fully connected layer is used to perform the multi-label classification task.

Figure 2 illustrates the architecture of LncLocFormer, which comprises four main components: (i) the embedding part, (ii) eight Transformer blocks, (iii) a localization-specific attention mechanism, and (iv) a fully connected layer that performs the multi-label classification task.

#### 2.2.1 Sequence embedding

Before feeding raw lncRNA sequences into a deep learning model, it is necessary to encode them as numeric vectors. The two most commonly used coding techniques are *k*-mer coding and one-hot coding. However, *k*-mer coding loses the sequence order information, while one-hot coding ignores the relationship between different nucleotides. In order to tackle these limitations, we employed an effective subsequence embedding method (Zeng *et al.* 2022), which can preserve the sequence order information of lncRNAs and reflect the relationship between different *k*-mers. The main idea of the subsequence embedding method is to split a lncRNA sequence into a number of consecutive, non-overlapping subsequences. Then, we extracted patterns from each subsequence and combined these patterns to obtain a complete representation of the lncRNA sequence.

Specifically, the subsequence embedding method involves several steps. First, we split a lncRNA sequence into *n* consecutive, non-overlapping subsequences. Then, we used an embedding technique to encode each subsequence. Word2vec is a popular word embedding technique in NLP that has demonstrated potential in many bioinformatics tasks (Wu *et al.* 2022, Li *et al.* 2023). Thus, we pre-trained all lncRNA sequences in our dataset to obtain the distribution representation of *k*-mers by using the word2vec method, and then used the distribution representation of *k*-mer features to represent these subsequences. In the training process, the parameter *k* was chosen from {1, 2, 3, 4, 5, 6} to find the best value. The skip-gram model was applied to maximize the co-occurrence likelihood function of the central word and corresponding context words. The other settings of word2vec were kept default to train word vectors. Finally, in our study, we set *k *=* *3, and the dimension of the word vector was 128. After pre-trained on the dataset, we obtained the word vectors and then combined these vectors to represent a lncRNA sequence. The whole subsequence embedding framework is shown in Supplementary Fig. S1. We refer to the original publication of the subsequence embedding method for more details (Zeng *et al.* 2022).

#### 2.2.2 Transformer blocks

So far, we have obtained the representation of a lncRNA sequence. The next step is to extract high-level features from the lncRNA representation. Transformer is a class of deep learning models that has achieved substantial breakthroughs in NLP and has recently been applied to various bioinformatics tasks. In our task, we employed eight Transformer blocks to model long-range dependencies within the lncRNA sequence and share information across the lncRNA sequence. The Transformer blocks are inspired by RealFormer, which is a state-of-the-art variant version of Transformer. The detailed structure is shown in the right side of Fig. 2. These Transformer blocks mainly consist of five components: relative positional encoding, multi-head attention, residual connection, “add & norm” component, and position-wise feed-forward network.

The first component is relative positional encoding. We know that Recurrent Neural Network (RNN) is a sequential structure that recurrently processes words one by one. Unlike RNN, the core part of Transformer is the attention mechanism. Using the attention mechanism to replace RNN loses the sequence order information, which causes that the model does not know the relative and absolute position information of each nucleotide in lncRNA sequences. Thus, it is necessary to add the sequence order information to assist the model in learning the position information. To use the sequence order information, we inject relative positional information by using relative positional encoding to the input representations. We borrowed the idea of traditional relative positional encoding and made some modifications. Specifically, the original formula of “traditional relative positional encoding” is as follows:

(1) $$P=x_{i}W_{q}{W_{k}}^{T}{x_{j}}^{T}+\beta_{ij}.$$

We made some modifications, resulting in the modified relative positional encoding formula to inject relative positional information:

(2) $$P=\alpha_{ij}x_{i}W_{q}{W_{k}}^{T}{x_{j}}^{T}+\beta_{ij},$$

where $\alpha_{ij}$ is a learnable parameter, $W_{q}$ and $W_{k}$ are learnable matrixes, and $\beta_{ij}$ is a relative position bias term. The main difference between the relative positional encoding and traditional relative positional encoding lies in the inclusion of the relative position scale term $\alpha_{ij}$. The advantage is, by adding this term, we can control the information passing for different relative locations more efficiently, making the model better able to capture the long-term dependencies within the lncRNA sequence.

After relative positional coding, LncLocFormer applies self-attention to learn the attention weights for each nucleotide pair in the lncRNA sequence. The attention weights can reveal the importance of sequence regions for subcellular localization. Specifically, for each input lncRNA sequence:

(3) $$\mathrm{lncRNA}=N_{1},N_{2},N_{3},\ldots ,N_{L-1},N_{L},$$

where *L* denotes the length of the lncRNA, *Nj* is one of the four nucleotide bases (A, C, G, and U) at the *j* position of the lncRNA sequence. Self-attention learns an attention score for each pair of nucleotides *i* and *j*. The attention score is computed by using a query $Q\in R^{d_{q}}$, a key $K\in R^{d_{k}}$, a value $V\in R^{d_{v}}$, a pre-softmax attention score Prev as follows:

(4) $$\mathrm{Attention}\left(Q,K,V\right)=\mathrm{Softmax}\left(\frac{QK^{T}}{\sqrt{d}}+\mathrm{Prev}\right)V,$$

where Prev indicates the attention scores from the previous self-attention layer.

Instead of using a single attention in the traditional Transformer architecture, computing attention scores using a set of queries, keys, and values enables the Transformer model to jointly attend to information at different positions, which is called the multi-head attention mechanism. In our study, the multi-head attention mechanism is applied to model long-range dependencies and share information across the lncRNA sequence. Each attention head*i* (*i *=* *1, 2, …, *H*) is computed as:

(5) $$\mathrm{hea}d_{i}=\mathrm{Attention}\left(QW_{i}^{Q},KW_{i}^{K},VW_{i}^{V},\mathrm{Pre}v_{i}\right),$$

where $W_{i}^{Q}$, $W_{i}^{K}$, and $W_{i}^{V}$ are learnable parameter matrixes, Prev*i* is the slice of Prev corresponding to head*i*.

Here, each attention head is independent. All head*i* are concatenated and transformed with another linear projection to obtain the final multi-head output values.

(6) $$\mathrm{Multi}-\mathrm{head}=\mathrm{Concat}\left(\mathrm{hea}d_{1},\;\mathrm{hea}d_{2},\ldots ,\mathrm{hea}d_{H}\right)W^{O},$$

where $W^{O}$ is a learnable parameter matrix and *H* is the number of heads. Based on the multi-head attention mechanism, each head may attend to different parts of the input lncRNA sequence.

In addition to relative positional coding and multi-head attention, the Transformer block has a standard architecture, which includes residual connection, “add & norm” component, and position-wise feed-forward network. Specifically, the queries, keys, and values are derived from the outputs of the previous Transformer block, a residual connection is employed to avoid gradient vanishing or gradient exploding problems. The “add & norm” component has two operations: addition and layer normalization. This addition operation from the residual connection is immediately followed by layer normalization. The position-wise feed-forward network transforms the representation at all the sequence positions using a fully connected layer.

#### 2.2.3 Localization-specific attention

The standard attention mechanisms in Transformer blocks only tell us which nucleotides are considered very important for the overall prediction. However, lncRNA subcellular localization is a dynamic process, which is treated as a multi-label classification problem in our study. Therefore, it would be more informative to analyze which nucleotides are considered important for each subcellular localization compartment. With this motivation, we designed a localization-specific attention mechanism after the Transformer blocks.

In the study, we have four subcellular localization compartments (nucleus, cytoplasm, chromatin, and insoluble cytoplasm). We used the multi-head attention mechanism to obtain the weight (importance) of every nucleotide in one subcellular localization compartment. Then, we repeated the process four times (here, four represents the number of subcellular localization compartments). As a result, we obtained four kinds of attention scores for four subcellular localization compartments. Specifically, we trained four attention matrices $\alpha_{1},\alpha_{2},\alpha_{3},\alpha_{4}\epsilon R^{n\times 1}$ for each subcellular localization compartment.

(7) $$\alpha_{j}=\mathrm{softmax}\left(VW_{\alpha }^{j}+b_{\alpha }^{j}\right),j=1,2,3,4,$$

where $V\in R^{n\times d}$ is the representation of the lncRNA obtained by the Transformer blocks and $W_{\alpha }^{j}\in R^{d\times 1},b_{\alpha }^{j}\in R$ are the learnable weight matrix and bias term. After that, we used $\alpha_{j}$ to aggregate V under label j and used a dense layer with sigmoid activation function to obtain the localization probability:

(8) $${\hat{s}}^{j}=\mathrm{sigmoid}\left(\alpha_{j}^{T}VW_{s}+b_{s}\right).$$

Finally, the $\left\{{\hat{s}}^{j}|j=1,2,3,4\right\}$ is used as the final prediction, and the cross-entropy loss is computed by ${\hat{s}}^{j}$ and $y^{j}$ to perform gradient descent.

Overall, the benefits of the localization-specific attention mechanism can be summarized as follows:

1. The localization-specific attention mechanism is a fine-grained interpretability technique that can provide support for the interpretability of each subcellular localization.
2. The localization-specific attention mechanism tends to be less heavily biased toward the most frequent compartments, which alleviates the imbalance data distribution problem.
3. The localization-specific attention learns multiple attention scores and uses them for prediction, resulting in more accurate and robust results.

Finally, in the classification part, a fully connected layer is applied to perform the multi-label classification task.

### 2.3 Deep learning baseline models and existing predictors

In this study, we focus on constructing powerful deep learning models to predict lncRNA subcellular localizations. To demonstrate the effectiveness of LncLncFormer, we compared it with several deep learning baseline models.

1. *k*-mer + MLP, this model extracts *k*-mer frequency features, which are fed into a MLP layer to output subcellular localizations.
2. Word2vec + MLP, this model encodes lncRNA sequences by using the word2vec technique, followed by feeding the sequence representation to a MLP layer for subcellular localization prediction.
3. Word2vec + CNN + MLP, this model converts lncRNA sequences to embedding vectors learned by the word2vec technique, followed by a CNN layer, then uses a MLP layer to predict the subcellular localizations.
4. Word2vec + Bi-LSTM + MLP, this model converts lncRNA sequences to embedding vectors learned by the word2vec technique, followed by a Bi-LSTM layer, then uses a MLP layer to predict the subcellular localizations.
5. Glove + MLP, this model encodes lncRNA sequences by using the Glove technique, followed by feeding the sequence representation to a MLP layer for subcellular localization prediction.
6. Glove + CNN + MLP, this model converts lncRNA sequences to embedding vectors learned by the Glove technique, followed by a CNN layer, then uses a MLP layer to predict the subcellular localizations.
7. Glove + Bi-LSTM + MLP, this model converts lncRNA sequences to embedding vectors learned by the Glove technique, followed by a Bi-LSTM layer, then uses a MLP layer to predict the subcellular localizations.

In the study, we used grid search to find the optimal parameters for these deep learning baseline models.

To further evaluate the performance of LncLocFormer in predicting lncRNA subcellular localizations, we compared LncLocFormer with several existing state-of-the-art predictors by using a hold-out test set. We selected lncLocator (Cao *et al.* 2018), iLoc-lncRNA (Su *et al.* 2018), Locate-R (Ahmad *et al.* 2020), DeepLncLoc (Zeng *et al.* 2022), iLoc-LncRNA(2.0) (Zhang *et al.* 2022), and GraphLncLoc (Li *et al.* 2023) as the compared predictors. LncLocator and DeepLncLoc can predict five types of subcellular localizations, including nucleus, cytoplasm, cytosol, ribosome, and exosome. iLoc-lncRNA, Locate-R, iLoc-LncRNA(2.0), and GraphLncLoc can predict four types of subcellular localizations, including nucleus, cytoplasm, ribosome, and exosome. We did not compare LncLocFormer with lncLocator 2.0 since lncLocator 2.0 only provides the predicted CNRCI values instead of probabilities.

### 2.4 Evaluation metrics

To evaluate the performance of LncLocFormer with deep learning baseline models, we selected some evaluation metrics which are widely used in multi-label classification problem (Li *et al.* 2019). These evaluation metrics include average *F*-measure (Ave-*F*1), micro precision (MiP), micro recall (MiR), micro *F*-measure (MiF), and each area under receiver operating characteristic curve (AUC) for each subcellular localization. For convenience, $y_{i}^{j},{\hat{y}}_{i}^{j}\in 0,1$ are the ground truth and predicted value of lncRNA i for subcellular localization j, respectively, and ${\hat{y}}_{i}^{j}=1$ if ${\hat{s}}^{j}>0.5$, otherwise ${\hat{y}}_{i}^{j}=0$.

Ave-*F*1 is the harmonic mean of average precision and average recall, which is used in the CAFA challenge (Zhang *et al.* 2019), a protein function prediction challenge. We compute Ave-*F*1 using the following formulas:

(9) $$\mathrm{Ave}-F1=\frac{2\times \mathrm{AvgPre}\left(t\right)\times A\mathrm{vgRec}\left(t\right)}{\mathrm{AvgPre}\left(t\right)+\mathrm{AvgRec}\left(t\right)},$$

(10) $$\mathrm{AvgPre}\left(t\right)=\frac{1}{m\left(t\right)}\times \sum_{i=1}^{m\left(t\right)}\mathrm{pr}e_{i}\left(t\right),$$

(11) $$\mathrm{AvgRec}\left(t\right)=\frac{1}{N}\times \sum_{i=1}^{N}\mathrm{re}c_{i}\left(t\right),$$

where

$${\mathrm{pre}}_{i}\left(t\right)=\frac{\sum_{j}y_{i}^{j}\times {\hat{y}}_{i}^{j}}{\sum_{j}{\hat{y}}_{i}^{j}},{\mathrm{rec}}_{i}\left(t\right)=\frac{y_{i}^{j}\times {\hat{y}}_{i}^{j}}{\sum_{i}y_{i}^{j}}.$$

MiF is the harmonic mean of MiP and MiR, which is used in the BioASQ challenge (You *et al.* 2021), a challenge on large-scale biomedical semantic indexing and question answering. It is defined as follows:

(12) $$\mathrm{MiF}=\frac{2\times \mathrm{MiP}\times \mathrm{MiR}}{\mathrm{MiP}+\mathrm{MiR}},$$

where

$$\mathrm{MiP}=\frac{\sum_{j=1}^{M}\sum_{i=1}^{N}y_{i}^{j}\times {\hat{y}}_{i}^{j}}{\sum_{j=1}^{M}\sum_{i=1}^{N}{\hat{y}}_{i}^{j}},\;\mathrm{MiR}=\frac{\sum_{j=1}^{M}\sum_{i=1}^{N}y_{i}^{j}\times {\hat{y}}_{i}^{j}}{\sum_{j=1}^{M}\sum_{i=1}^{N}y_{i}^{j}}.$$

Considering that the existing predictors are designed as multi-class predictors rather than multi-label predictors, to evaluate the performance of LncLocFormer with existing predictors, we evaluated the performance from two perspectives: the multi-label and the multi-class perspectives.

In the multi-label perspective, we used Precision@k (P@k, which represents the number of correct predictions over *k*) to evaluate the performance (Zhang *et al.*). It is defined as follows:

(13) $$P@k=\frac{1}{N}\sum_{i=1}^{N}\frac{1}{k}\sum_{j\mathrm{ran}k_{k}\in \left({\hat{y}}_{i}^{*}\right)}y_{i}^{j},$$

where ${\mathrm{rank}}_{k}\left({\hat{y}}_{i}^{*}\right)$ returns the *k* largest indices of ${\hat{y}}_{i}^{*}$ ranked in descending order. In the study, we only focus on the subcellular localization with the highest probability, thus, *k* is set to 1.

In the multi-class perspective, consistent with the current state-of-the-art lncRNA subcellular localization predictors, we used Accuracy (ACC), Macro F-measure (MaF), Macro Precision (MaP), Macro Recall (MaR), and AUC as evaluation metrics.

(14) $$\mathrm{ACC}=\frac{1}{N}\sum_{i=1}^{N}\sum_{j=1}^{M}y_{i}^{j}\times {\hat{y}}_{i}^{j},$$

(15) $$\mathrm{Ma}P_{j}=\frac{1}{N}\sum_{i=1}^{N}\frac{y_{i}^{j}\times {\hat{y}}_{i}^{j}}{{\hat{y}}_{i}^{j}},$$

(16) $$\mathrm{Ma}R_{j}=\frac{1}{N}\sum_{i=1}^{N}\frac{y_{i}^{j}\times {\hat{y}}_{i}^{j}}{y_{i}^{j}},$$

(17) $$\mathrm{MaF}=\frac{1}{M}\sum_{j=1}^{M}\frac{2\times \mathrm{Ma}P_{j}\times \mathrm{Ma}R_{j}}{\mathrm{Ma}P_{j}+\mathrm{Ma}R_{j}}.$$

### 2.5 Implementation details

LncLocFormer is implemented using PyTorch (Paszke *et al.*). A grid search strategy was employed to find the optimal parameters of LncLocFormer, and Supplementary Table S1 provides a summary of the optimal hyper-parameters and the corresponding search space. The used loss function is the cross-entropy function. The skip-gram model (Mikolov *et al.*) is used to pre-train the *k*-mer embedding vectors. We used the Adam optimizer with a learning rate of 0.0003. The learning rate is warm-uped over the first four epochs and decayed linearly for the remaining training steps. The batch size is set to 64. In the Transformer blocks, we used eight heads and hidden size of 128. To prevent the low-rank bottleneck, we enhanced the size of query/key/value into 64 using a dense layer (Bhojanapalli *et al.*). We keep at most 8196 nt for each lncRNA and divide them into 512 subsequences by using the subsequence embedding method. The dropout rate is set to 0.2 for the embedding layer and 0.1 for other layers. The maximum relative distance in the position embedding is set to 25.

## 3 Results

### 3.1 Comparison with deep learning baseline models

In this section, we investigated the effectiveness of LncLocFormer (subsequence embedding + Transformer blocks + localization-specific attention + MLP). We conducted 5-fold CV to evaluate the performance of LncLocFormer with other deep learning baseline models. In particular, we split the benchmark dataset into a training set (90%) and a hold-out test set (10%). Next, we performed 5-fold CV by further splitting the training set into 80% training and 20% validation. The process was repeated five times, and the final prediction results were the average of five validation results. The performances of LncLocFormer and other deep learning baseline models using 5-fold CV are shown in Table 1. We can observe that LncLocFormer outperforms other deep learning baseline models, except for the MiP. Specifically, LncLocFormer obtains Ave-*F*1 of 0.719, MiR of 0.721, MiF of 0.701, and average AUC of 0.648, while GloVe + Bi-LSTM + MLP obtains the best MiP (0.712). These observations indicate the superiority of LncLocFormer network architecture.

**Table 1.** Performance of LncLocFormer and other deep learning baseline models using 5-fold CV.^a^

Model	Ave-*F*1	MiP	MiR	MiF	AUC				
					Nucleus	Cytoplasm	Chromatin	Insoluble cytoplasm	Average
K-mer + MLP	0.670	0.651	0.645	0.648	0.680	0.636	0.616	0.582	0.629
Word2vec + MLP	0.690	0.664	0.660	0.662	0.660	0.580	0.561	0.526	0.582
Word2vec + CNN + MLP	0.697	0.692	0.652	0.672	0.679	0.591	0.610	0.582	0.616
Word2vec + Bi-LSTM + MLP	0.689	0.670	0.653	0.661	0.682	0.595	0.597	0.622	0.624
GloVe + MLP	0.697	0.685	0.656	0.670	0.662	0.560	0.588	0.574	0.596
GloVe + CNN + MLP	0.697	0.674	0.677	0.675	0.668	0.636	0.580	0.541	0.606
GloVe + Bi-LSTM + MLP	0.679	**0.712**	0.602	0.652	0.676	0.573	0.570	0.510	0.582
LncLocFormer	**0.719**	0.683	**0.721**	**0.701**	**0.686**	**0.651**	**0.623**	**0.632**	**0.648**

a The best performance values are highlighted in bold.

### 3.2 Comparison with existing predictors

In the previous section, we performed 5-fold CV to obtain the best parameters and evaluated the performance of LncLocFormer with other deep learning baseline models. To further evaluate the performance of LncLocFormer in predicting lncRNA subcellular localizations, we compared LncLocFormer with several existing state-of-the-art predictors by using a hold-out test set. In particular, we selected the current predictors follow these criteria: (i) the availability of web server or stand-alone version; (ii) input that only needs lncRNA sequences; and (iii) outputs that include predictive probabilities for subcellular localization. Finally, we used the following web servers for comparison: lncLocator (http://www.csbio.sjtu.edu.cn/bioinf/lncLocator/), iLoc-lncRNA (http://lin-group.cn/server/iLoc-LncRNA/), Locate-R (http://locate-r.azurewebsites.net), DeepLncLoc (http://bioinformatics.csu.edu.cn/DeepLncLoc/), iLoc-LncRNA(2.0) (http://lin-group.cn/server/iLoc-LncRNA(2.0)/), and GraphLncLoc (http://csuligroup.com:8000/GraphLncLoc/). The detailed prediction results on the hold-out test set are shown in Supplementary Table S2. The P@1 of LncLocFormer and existing predictors on the hold-out test set is shown in Table 2.

**Table 2.** P@1 of LncLocFormer and existing predictors on the hold-out test set (RNALocate v2.0).^a^

Predictor	P@1
lncLocator	0.232
iLoc-LncRNA	0.348
Locate-R	0.275
DeepLncLoc	0.304
iLoc-LncRNA(2.0)	0.333
GraphLncLoc	0.536
LncLocFormer	**0.899**

a The best performance values are highlighted in bold.

From Table 2, we can observe that LncLocFormer significantly outperforms existing predictors. Specifically, LncLocFormer obtains P@1 of 0.899, which is much higher than that of lncLocator (0.232), iLoc-LncRNA (0.348), Locate-R (0.275), DeepLncLoc (0.304), iLoc-LncRNA(2.0) (0.333), and GraphLncLoc (0.536). These results demonstrate that LncLocFormer has a powerful ability in predicting multi-label lncRNA subcellular localizations and achieves state-of-the-art performance on the hold-out test set. However, a natural question arises: why is there such a significant gap between LncLocFormer and the other predictors? We believe that it is impossible to achieve this by relying only on model architecture. One of the most possible explanations is that the used datasets are different. lncLocator, iLoc-LncRNA, Locate-R, DeepLncLoc, iLoc-LncRNA(2.0), and GraphLncLoc all used the RNALocate v1.0 database (Zhang *et al.* 2017) to train and test their models, while LncLocFormer is trained and tested by using the RNALocate v2.0 database (Cui *et al.* 2022). Therefore, we believe that the difference between the two datasets is the main reason for the large gap between LncLocFormer and other predictors.

To investigate the difference between the two datasets, we plotted the distributions of the RNALocate v1.0 dataset used in the six predictors and the RNALocate v2.0 dataset used in LncLocFormer, as shown in Supplementary Fig. S2. By comparing Supplementary Fig. S2a with b, we can observe that there are significant differences between the RNALocate v2.0 and RNALocate v1.0 datasets. For example, in the RNALocate v2.0 dataset, the number of lncRNAs located in the nucleus is much greater than the number of lncRNAs located in the cytoplasm. In contrast, in the RNALocate v1.0 dataset, the number of lncRNAs located in the cytoplasm is slightly larger than the number of lncRNAs located in the nucleus. These findings demonstrate that the data from the two datasets are not independently identical distributed (i.i.d.).

To make a fairer comparison and to prove the superiority of LncLocFormer architecture, we used the RNALocate v1.0 database to retrain our model. Specifically, we employed the same training set and test set utilized in our previous predictor, DeepLncLoc, to retrain and test LncLocFormer. Since the dataset is generated for the multi-class prediction problem, we used a softmax activation function to replace the sigmoid activation function in the final fully connected layer to perform the multi-class prediction task. As with previous studies, we used ACC, MaF, MaP, and MaR as evaluation metrics to evaluate LncLocFormer and the existing predictors. The detailed prediction results on the RNALocate v1.0 test set are shown in Supplementary Table S3. The performance of LncLocFormer (using the RNALocate v1.0 dataset for training and test) and the existing predictors is shown in Table 3. In Table 3, the evaluation metrics we pay most attention to are MaF and ACC. From Table 3, we can observe that LncLocFormer still outperforms existing predictors in terms of MaF and ACC.

**Table 3.** Performance comparison of LncLocFormer (using the RNALocate v1.0 dataset for training and test) with the existing predictors.^a^

Predictor	MaP	MaR	MaF	ACC
lncLocator	0.288	0.292	0.276	0.433
iLoc-lncRNA	0.488	0.445	0.458	0.507
Locate-R	0.374	0.317	0.329	0.403
DeepLncLoc	0.680	0.543	0.563	0.537
iLoc-LncRNA(2.0)	0.460	0.384	0.390	0.433
GraphLncLoc	**0.731**	0.549	0.560	0.522
LncLocFormer	0.696	**0.566**	**0.597**	**0.612**

a The best performance values are highlighted in bold.

### 3.3 Motif analysis

In the study, we designed a localization-specific attention mechanism to obtain distinct attention weights for each subcellular localization and find the most likely motifs in lncRNA sequences. To investigate the performance of localization-specific attention mechanism in LncLocFormer, we conducted some motif analyses.

**Figure 3.**
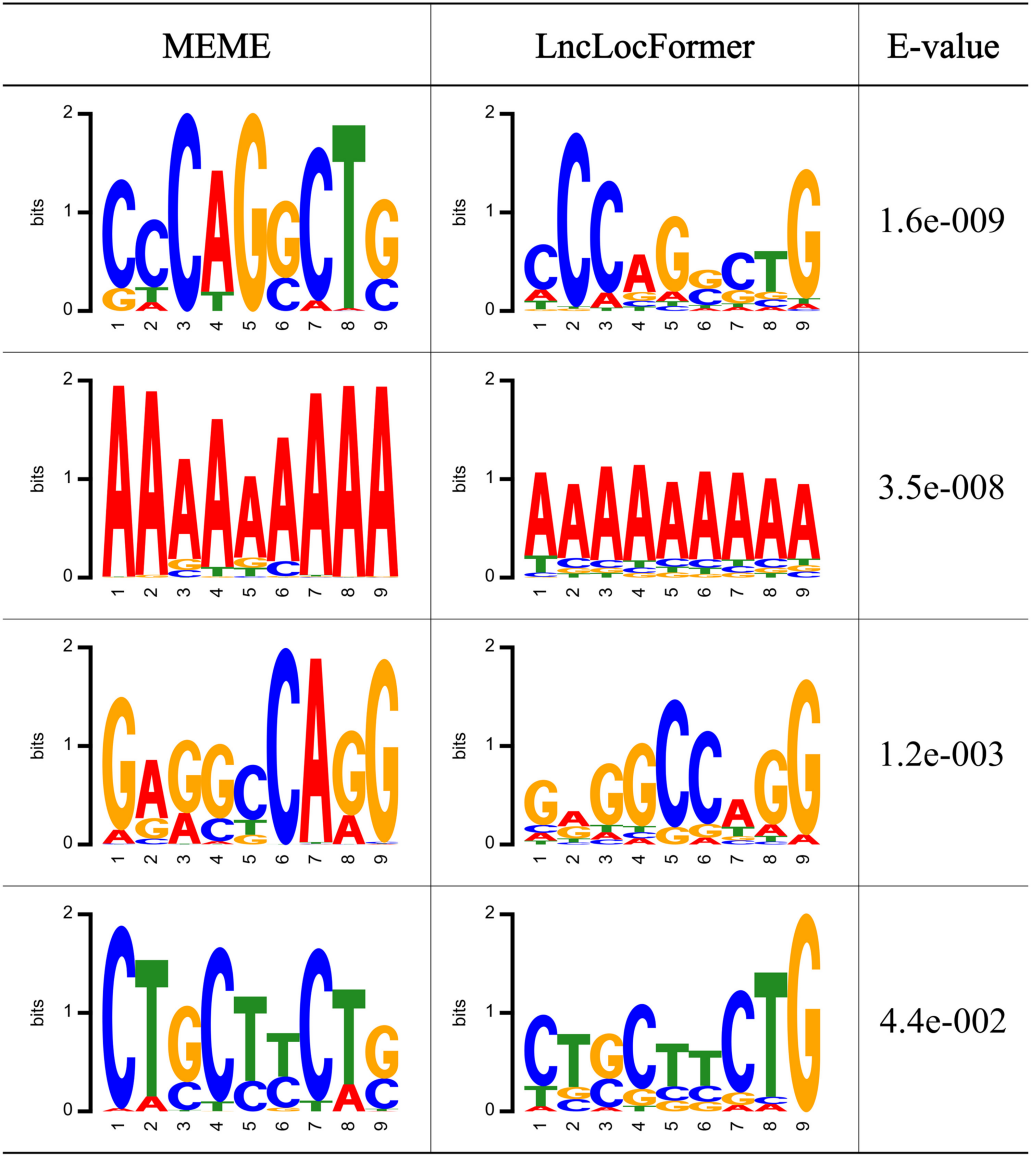
Motifs discovered by MEME suite (left) and by LncLocFormer (middle). The right are the *E*-values of the motifs found by the MEME suite.

First, we tested whether LncLocFormer could find the most frequently recurring motifs. In particular, we used the MEME suite (Bailey *et al.* 2009) to find the motifs in our dataset. The motifs are analyzed with the width of nine, and the *E*-value is set to 0.05. We used a threshold to determine the importance of attention weights. The threshold is set to the multiplicative inverse of the input sequence length. Because if the attention weights on the lncRNA sequence are randomly distributed, the mathematic expectation of all attention weights on the lncRNA sequence is the multiplicative inverse of the input sequence length. Only if the attention weight of a nucleotide is larger than the mathematic expectation, we believe that LncLocFormer pays attention to the nucleotide. If the attention weight of a nucleotide is smaller than the mathematic expectation, we believe that LncLocFormer does not focus on the nucleotide. Figure 3 displays the representative examples, with the left column depicting the motifs found by the MEME suite, the middle column showing the motifs discovered by LncLocFormer, and the right column displaying the *E*-values of the motifs found by the MEME suite. From Fig. 3, we can observe that LncLocFormer can capture the motifs that are similar to those found by the MEME suite, which means LncLocFormer can capture the most frequently recurring motifs.

**Figure 4.**
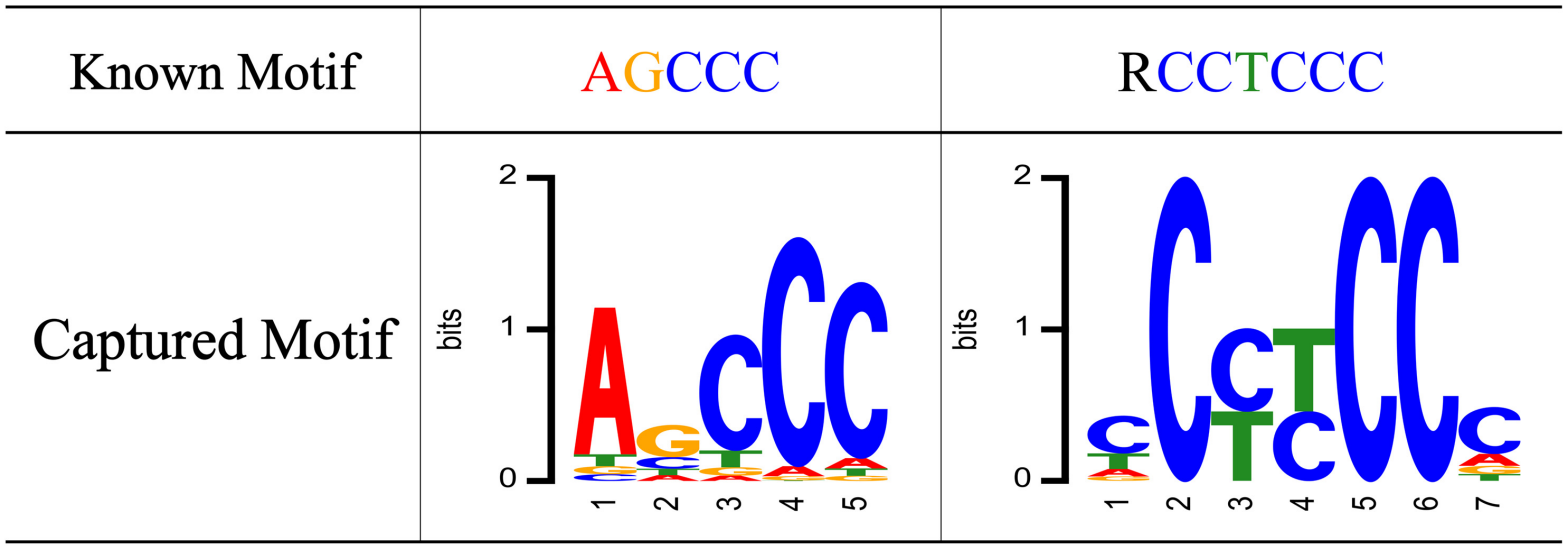
LncLocFormer captures two known motifs, which are related to nucleus localization.

Second, we investigated whether LncLocFormer could capture some known motifs. Specifically, we searched for some known motifs in recent literature that are related to subcellular localization. Lubelsky *et al.* (Lubelsky and Ulitsky 2018) found that the repeated motif RCCTCCC (where R denotes A/G) drives lncRNAs to be located in the nucleus. Zhang *et al.* (2014) identified the motif AGCCC act as a general nucleus localization signal. We used motifs RCCTCCC and AGCCC as examples to show the performance of LncLocFormer. The captured motifs by LncLocFormer for nucleus are shown in Fig. 4. From Fig. 4, we can observe that LncLocFormer can capture motifs that are similar to those that are already known.

### 3.4 Case study

**Figure 5.**
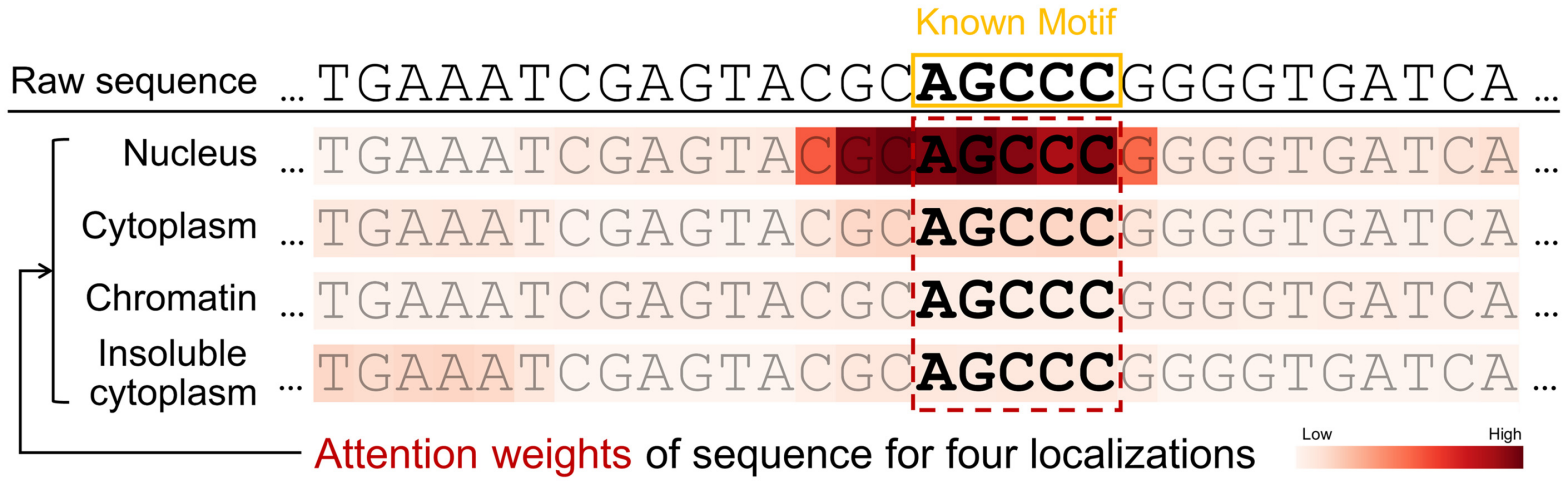
Attention weight visualization of lncRNA Cerox1.

To better understand the role of the localization-specific attention, we visualized the attention matrices $\alpha_{1}$, $\alpha_{2}$, $\alpha_{3}$, ${\mathrm{and}\;\alpha }_{4},$ which are computed by Equation (7) and gave a case study in Fig. 5 using lncRNA Cerox1 (cytoplasmic endogenous regulator of oxidative phosphorylation 1, NCBI ID: 115804232) as an example. The true label of lncRNA Cerox1 is nucleus. According to Zhang *et al.* (2014), the motif AGCCC act as a general nucleus localization signal. We obtained four kinds of attention weights of different subcellular localizations, and highlighted the sequence using different degrees of red based on the values of attention weights. From Fig. 5, we can observe that the attention matrix of nucleus captures an important region containing the core motif (AGCCC), while the attention matrices of other subcellular localizations fail to capture the motif AGCCC. Although LncLocFormer cannot find the exact known motifs, it can capture motifs that are very similar to the known motifs. The results suggest the potential of LncLocFormer in motif discovery.

### 3.5 Ablation study

In order to discover the essential components of LncLocFormer, we conducted an ablation study by removing individual parts of LncLocFormer. In particular, we tested the model without localization-specific attention and the model without positional encoding. The former model lost multiple attention weights for different subcellular localizations, while the latter model lost the sequence order information. The results are shown in Table 4. In Table 4, the evaluation metrics we pay most attention to are Ave-*F*1, MiF, and average AUC. From Table 4, we can observe that localization-specific attention is the most important part of LncLocFormer. Without localization-specific attention, Ave-*F*1, MiF, and average AUC decrease from 0.719, 0.701, and 0.648 to 0.627, 0.591, and 0.614, respectively. Additionally, positional encoding is also useful in LncLocFormer. Without positional encoding, Ave-*F*1, MiF, and average AUC decrease from 0.719, 0.701, and 0.648 to 0.710, 0.691, and 0.643, respectively. The results confirm the effectiveness of localization-specific attention and positional encoding in LncLocFormer.

**Table 4.** The performances of various models in the ablation study.^a^

Model	Ave-*F*1	MiP	MiR	MiF	AUC				
					Nucleus	Cytoplasm	Chromatin	Insoluble cytoplasm	Average
Without localization-specific attention	0.627	0.550	0.640	0.591	**0.688**	0.638	0.599	0.532	0.614
Without positional encoding	0.710	0.653	**0.734**	0.691	0.672	**0.660**	0.611	0.628	0.643
LncLocFormer	**0.719**	**0.683**	0.721	**0.701**	0.686	0.651	**0.623**	**0.632**	**0.648**

a The best performance values are highlighted in bold.

In addition, we observed an interesting phenomenon, i.e. models without localization-specific attention can produce better results for the nucleus localization, while models without positional encoding can produce better results for the cytoplasm localization. Regarding the better results for nucleus localization with models lacking localization-specific attention, one possible explanation is that the benchmark dataset is imbalanced. The nucleus localization has a larger number of samples compared to the other three classes. When localization-specific attention is removed, the model may exhibit a bias toward the classes with more samples because this can lead to higher overall accuracy. This bias could result in improved predictions for the nucleus localization while leading to poorer predictions for the other three subcellular localizations. As for the better results for cytoplasm localization with models lacking positional encoding, the possible reason is that the lncRNA sequences belonging to the cytoplasm localization in the benchmark dataset are often quite long. In very long sequences, the relative position encoding may not effectively capture the relative distance relationship between nucleotides and may forget what has been learned in the sequence. Instead, the addition of relative position encoding may introduce noise or unnecessary information, resulting in a decline in prediction performance for the cytoplasm localization.

### 3.6 Web server

To facilitate the use of LncLocFormer, we developed a user-friendly web server, http://csuligroup.com:9000/LncLocFormer. LncLocFormer requires lncRNA sequences with more than 200 and <10 000 nucleotides as input. Users can paste the lncRNA sequence into the input box and click on the submit button to see the predicted results. For each lncRNA sequence, the predicted probabilities and attention weights for each subcellular localization are displayed on the screen. In general, LncLocFormer takes <10 s to predict the subcellular localization of a given lncRNA sequence. We believe that LncLocFormer is a convenient and efficient tool in the field of lncRNA subcellular localization prediction.

## 4 Conclusion

In the study, we proposed LncLocFormer, a multi-label lncRNA subcellular localization predictor that utilizes Transformer and localization-specific attention mechanism. Unlike many previous computational methods that only consider a single subcellular localization for a lncRNA sequence, LncLocFormer can predict multiple subcellular localizations simultaneously for each lncRNA sequence. Due to the uncertainty of the number of labels for each lncRNA sequence and the implicit relationship between the labels, the multi-label classification problem is more complicated than conventional multi-class classification tasks. By using Transformer blocks and localization-specific attention mechanism, LncLocFormer can predict lncRNA multiple subcellular localizations accurately, learn different motifs for each subcellular localization, and capture some motifs that are very similar to known motifs. Our extensive experimental results demonstrate that LncLocFormer outperforms existing state-of-the-art predictors. We believe that LncLocFormer can serve as a useful tool for predicting lncRNA multiple subcellular localizations.

Although LncLocFormer shows promising results, there are some limitations that may influence the performance of LncLocFormer. The performance of LncLocFormer is limited by the number of samples in the RNALocate dataset. In the study, we only have 811 samples for the multi-label classification task. With lncRNA subcellular localization becoming a more important research topic, we could obtain more reliable data that can be used for training and test. Alternatively, we could consider transferring some data from other domains to aid in the research topic.

Furthermore, LncLocFormer utilizes eight Transformer blocks and localization-specific attention, resulting in a lot of parameters that need to be tuned. Consequently, the training time of LncLocFormer is very long. With the development of deep learning techniques, more and more advanced knowledge distillation and network pruning techniques will be proposed. As a result, using a lightweight network architecture to predict lncRNA subcellular localization is a promising future direction.

## Supplementary Material

btad752_Supplementary_Data
