# Supplementary material for: LncLocFormer: a Transformer-based deep learning model for multi-label lncRNA subcellular localization prediction by using localization-specific attention mechanism
Source: Bioinformatics. 2023 Dec 18;39(12):btad752. doi: 10.1093/bioinformatics/btad752 (PMC10749772; doi:10.1093/bioinformatics/btad752)
Supplement: btad752_Supplementary_Data [file btad752_supplementary_data.zip › Supplementary materials_20231111.pdf]

Supplementary Materials

LncLocFormer: a Transformer-based deep learning model for multi-label lncRNA subcellular localization prediction by using localization-specific attention mechanism

Min Zeng[1], Yifan Wu[1], Yiming Li[1], Rui Yin[2], Chengqian Lu[3], Junwen Duan[1], and Min Li[1,*]

[1]School of Computer Science and Engineering, Central South University, Changsha, Hunan, 410083, China.

[2]Department of Health Outcomes and Biomedical Informatics, University of Florida, Gainesville, Florida, 32603, USA.

[3]School of Computer Science, Key Laboratory of Intelligent Computing and Information Processing, Xiangtan University, Xiangtan, Hunan, 411105, China.

*Corresponding author: Min Li, E-mail: limin@mail.csu.edu.cn

Supplementary Figures

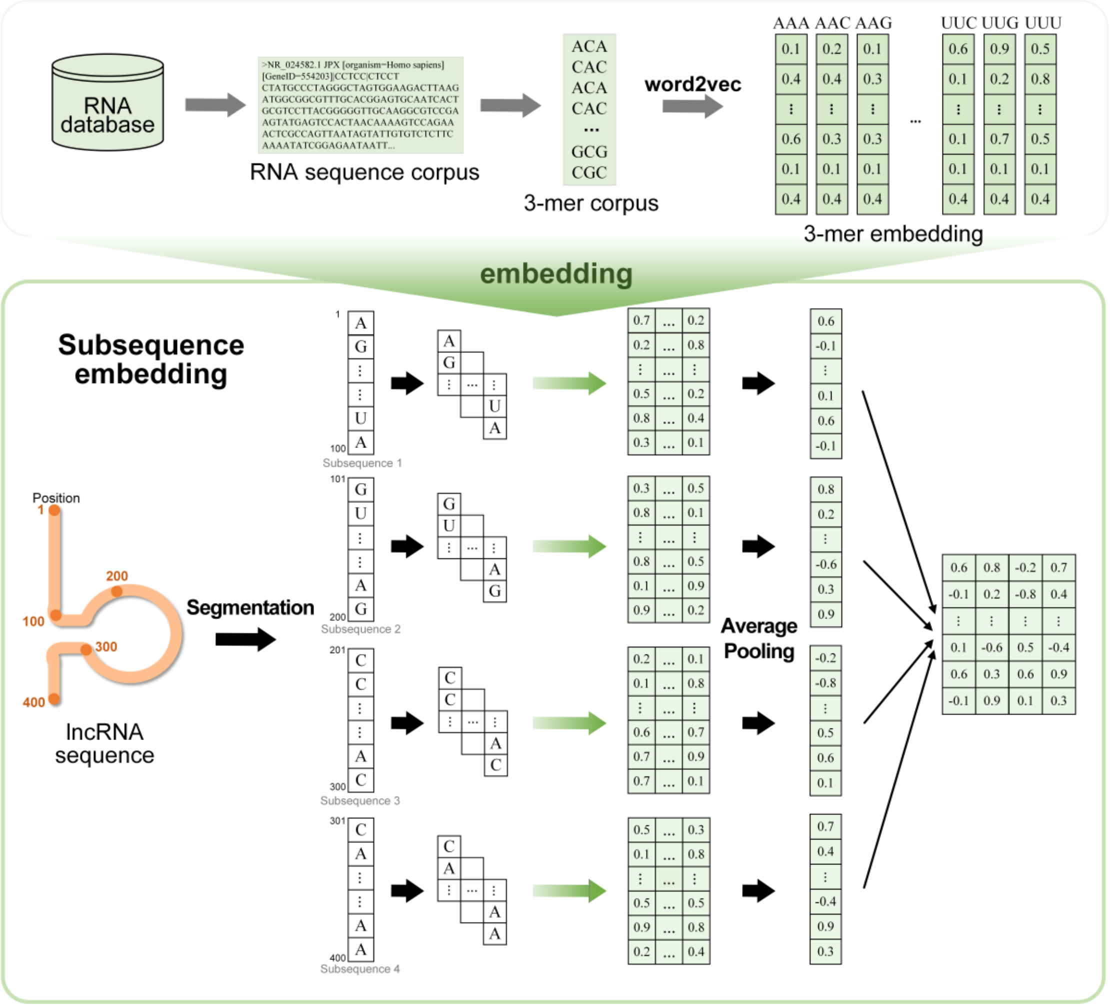

Supplementary Figure S1. Subsequence embedding framework. The figure is only an example. We first built the k-mer corpus, which consists of all k-mer sequences built by splitting lncRNA sequences. Then, we learned the representation vectors of k-mer of all lncRNA sequences. The input is a lncRNA sequence with a length of 400. The lncRNA sequence is split into 4 subsequences. According to the k-mer splitting of the lncRNA, we found the pre-trained vector of each k-mer, and then combined these vectors into a matrix as the representation of a subsequence. After subsequence embedding, we used an average pooling layer to extract the patterns of each subsequence. Then we combined these patterns together to obtain a matrix as the representation of the whole lncRNA sequence.

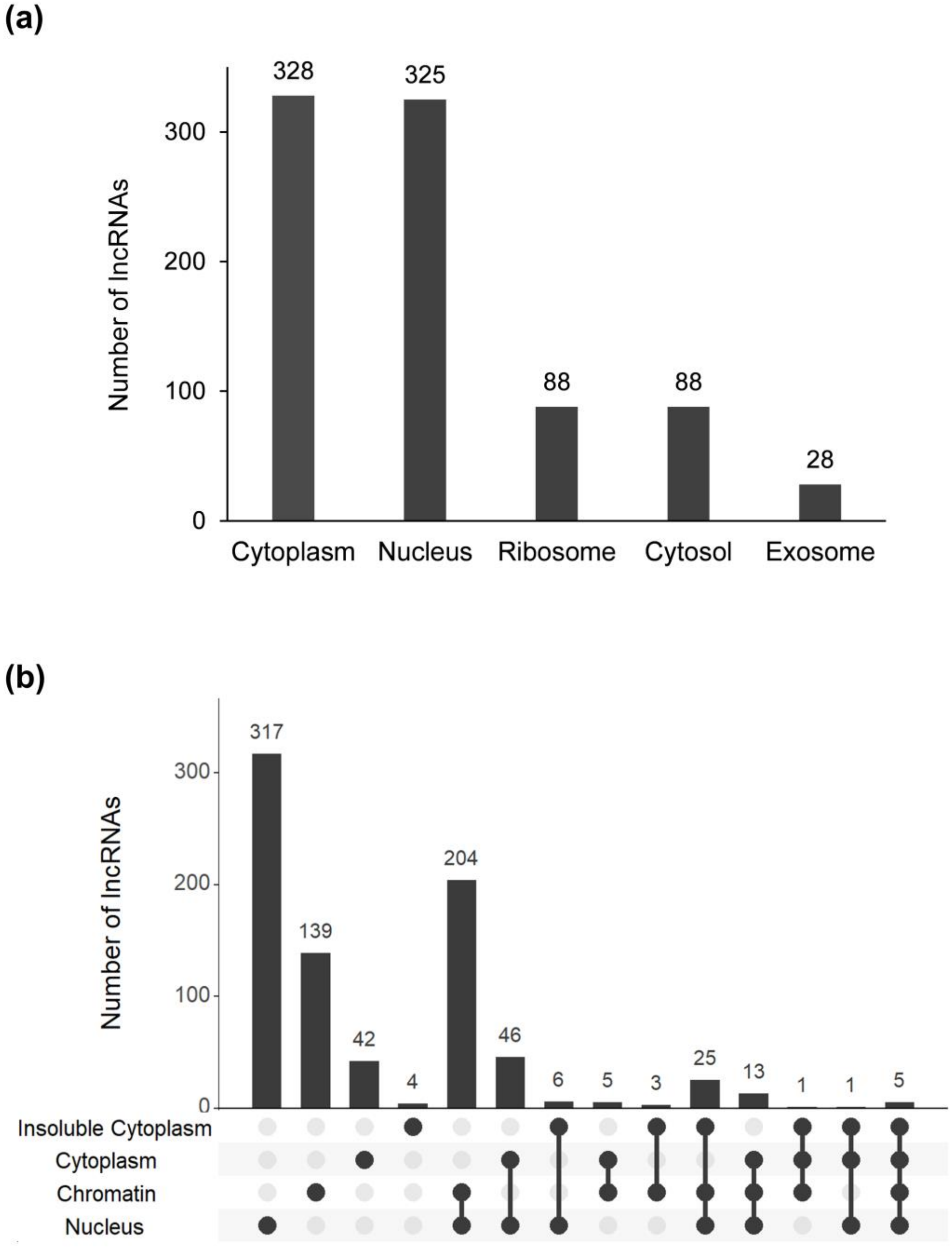

Supplementary Figure S2. The distributions of the RNALocate v1.0 and RNALocate v2.0 datasets, (a) RNALocate v1.0 dataset, (b) RNALocate v2.0 datasets.

Supplementary Table

Supplementary Table S1. The optimal hyper-parameters of LncLocFormer and the corresponding search space.

Hyper-parameters	Search space	Best value
k (-mer)	{1, 2, 3, 4, 5, 6}	3
The number of subsequences	{256, 512}	512
The dimension of the *k*-mer embedding using word2vec	{64, 128}	128
Learning rate	{0.0001, 0.0003, 0.00001}	0.0003
Batch size	{16, 32, 64, 128}	64
Dropout (embedding layer)	{0.1, 0.2, 0.3}	0.2
Dropout (other layer)	{0.1, 0.2, 0.3}	0.1

Supplementary Table Legend

Supplementary Table S2. The detailed prediction results of lncLocator, iLoc-lncRNA, Locate-R, iLoc-lncRNA 2.0, DeepLncLoc, GraphLncLoc, and LncLocFormer on the RNALocate 2.0 test set.

Supplementary Table S3. The detailed prediction results of lncLocator, iLoc-lncRNA, Locate-R, iLoc-lncRNA 2.0, DeepLncLoc, GraphLncLoc, and LncLocFormer on the RNALocate 1.0 test set.
